# Fourier transform infrared difference spectroscopy for studying the molecular mechanism of photosynthetic water oxidation

**DOI:** 10.3389/fpls.2013.00146

**Published:** 2013-05-21

**Authors:** Hsiu-An Chu

**Affiliations:** Institute of Plant and Microbial BiologyAcademia Sinica, Taipei, Taiwan

**Keywords:** photosystem II, oxygen evolution, FTIR, manganese cluster, infrared spectroscopy

## Abstract

The photosystem II reaction center mediates the light-induced transfer of electrons from water to plastoquinone, with concomitant production of O_2_. Water oxidation chemistry occurs in the oxygen-evolving complex (OEC), which consists of an inorganic Mn_4_CaO_5_ cluster and its surrounding protein matrix. Light-induced Fourier transform infrared (FTIR) difference spectroscopy has been successfully used to study the molecular mechanism of photosynthetic water oxidation. This powerful technique has enabled the characterization of the dynamic structural changes in active water molecules, the Mn_4_CaO_5_ cluster, and its surrounding protein matrix during the catalytic cycle. This mini-review presents an overview of recent important progress in FTIR studies of the OEC and implications for revealing the molecular mechanism of photosynthetic water oxidation.

## INTRODUCTION

**FIGURE 1:**
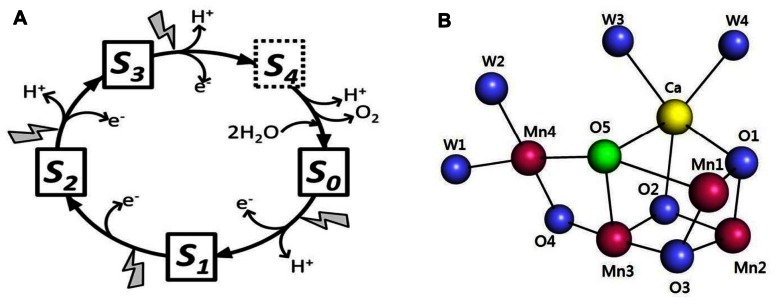
**(A)** S-state cycle of photosynthetic water oxidation. **(B)** Structure of the Mn_4_CaO_5_ cluster as revealed by the 1.9 Å XRD study (Umena et al., 2011). Images generated in the PyMOL program using Protein Data Bank entry 3ARC.

Photosynthetic water oxidation is catalyzed by a Mn_4_Ca cluster and its surrounding protein matrix in photosystem II (PSII; Ferreira et al., 2004; Loll et al., 2005; Yano et al., 2006; Umena et al., 2011). The oxygen-evolving complex (OEC) accumulates oxidizing equivalents from the photochemical reactions within PSII and cycles through five oxidation states, termed *S*_n_ (*n* = 0–4, *n* representing the storage of oxidizing equivalents in the OEC; Kok et al., 1970; **Figure 1A**). The molecule O_2_ is produced in the transition of S_3_-(S_4_)-S_0_. One of the recent major breakthroughs in PSII research was the report of the crystal structure of oxygen-evolving PSII at 1.9 Å resolution (Umena et al., 2011). The structure of the Mn_4_CaO_5_ cluster is shown in **Figure 1B**. Three Mn, one Ca, and four oxygen atoms form a cubane-like structure; the fourth Mn connects to the cubic structure by two μ-oxo-bridges. The Mn_4_CaO_5_ cluster is connected with four water molecules: two are ligated to Ca and two to Mn_4_ (Umena et al., 2011; **Figure 1B**). These water molecules are candidates for substrates in photosynthetic water oxidation. Another distinct feature in the structure is the apparently longer bond distances between the O_5_-bridging oxygen atom and neighboring metal ions, which indicates weak bonding of this oxygen atom in the cluster. O_5_ was proposed as a candidate for one of the substrates in dioxygen formation (Umena et al., 2011). More recent studies have suggested that the structure of the X-ray diffraction (XRD) model of PSII is modified by radiation-induced reduction of the Mn cluster (Luber et al., 2011; Grundmeier and Dau, 2012). Despite this problem, the 1.9 Å XRD structure is the crucial foundation for spectroscopic and mechanistic studies of photosynthetic water oxidation.

Several structural models of photosynthetic water oxidation have been proposed (Hoganson and Babcock, 1997; Pecoraro et al., 1998; McEvoy and Brudvig, 2006; Kusunoki, 2007; Pushkar et al., 2008; Siegbahn, 2009; Grundmeier and Dau, 2012), with differing locations and molecular structures of S-state intermediates (i.e., terminal water molecules or water-derived metal ligands). Several molecular spectroscopic techniques, including advanced electron paramagnetic resonance (EPR) spectroscopy (Britt et al., 2004; McConnell et al., 2011; Rapatskiy et al., 2012) and light-induced Fourier transform infrared (FTIR) difference spectroscopy (see below) have been extensively used to resolve this issue.

Fourier transform infrared difference spectroscopy has been widely used to study the structural changes in the OEC during the S-state catalytic cycle. The S_2_-minus-S_1_ mid-frequency (1800–1000 cm^-1^) FTIR difference spectrum was first reported in 1992 (Noguchi et al., 1992). The S_3_-minus-S_2_ spectrum of the PSII/OEC was reported in 2000 (Chu et al., 2000b), and spectra of flash-induced S-state transitions (S_1_ → S_2_, S_2_ → S_3_, S_3_ → S_0_, and S_0_ → S_1_) during the complete S-state cycle were reported 1 year later (Hillier and Babcock, 2001; Noguchi and Sugiura, 2001). Many FTIR studies of the OEC focused on the mid-frequency region (1800–1000 cm^-1^) of the IR spectrum, which contains information on structural changes of protein backbones and amino acid side-chains associated with S-state transitions of the OEC. One very important development in FTIR studies of the OEC were reports of high-frequency spectra (3700–3500 cm^-1^) of the OEC, which contain information on structural changes of the weakly H-bonded OH-stretching of active water molecules during S-state transitions of the OEC (Noguchi and Sugiura, 2000, 2002a,b). The other important developments were reports of low-frequency spectra (<1000 cm^-1^), which contain information on metal-ligand and manganese-substrate vibration modes of the OEC (Chu et al., 1999, 2000a,c; Yamanari et al., 2004; Kimura et al., 2005b).

This mini-review gives an overview of recent important progress in FTIR studies of the OEC, combined with new spectroscopic and XRD structural information, to understand the chemical mechanism of photosynthetic water oxidation. More comprehensive reviews on FTIR studies of the OEC are available (Noguchi, 2007, 2008a,b; Debus, 2008).

## OH-STRETCHING VIBRATIONAL MODES OF ACTIVE WATER MOLECULES IN THE HIGH-FREQUENCY REGION (3700–3500 cm^–1^) OF THE OEC

The reactions of substrate water during the S-state catalytic cycle of the OEC are of paramount importance to understand the chemical mechanism of photosynthetic water oxidation. In the new XRD structure of the Mn_4_CaO_5_ cluster, the four water molecules connected to the OEC are involved in a hydrogen-bonded network linking the Mn_4_CaO_5_-cluster and Y_Z_ (Umena et al., 2011). The bond distances (2.8–3.3 Å) between oxygen atoms of coordinated water molecules and their neighboring water molecules indicate that most of the O–H groups of the water molecules are weakly hydrogen bonded and will appear in the weakly hydrogen-bonded OH-stretch (3750–3500 cm^-1^) region of the FTIR spectra. Noguchi and colleagues reported flash-induced difference spectra of S-state transitions in the weakly H-bonded OH-stretching region (Noguchi and Sugiura, 2000, 2002a,b). One active water molecule on the OEC, which gave rise to the S_1_ band at ~3585 cm^-1^ and the S_2_ band at ~3618 cm^-1^, was identified at 250 K in light-induced S_2/_S_1_ FTIR difference spectrum (Noguchi and Sugiura, 2000) and during the S-state cycle at 10°C (Noguchi and Sugiura, 2002a,b) in PSII core complexes from *Thermosynechococcus elongatus*. The results indicated a weakened hydrogen bond of the OH group for one water molecule connected to the OEC during the S_1_ → S_2_ transition. In contrast to the S_1_ → S_2_ transition, the S_2_ → S_3_, S_3_ → S_0_, and S_0_ → S_1_ transitions all showed a negative OH-stretching mode at different frequencies, which indicates that these water (or hydroxide) molecules were involved in proton release reactions of the OEC or formed strong hydrogen-bonding interactions during these transitions (Noguchi and Sugiura, 2002a,b). In addition, these observations are consistent with a recent FTIR study which concluded that the proton release pattern from the substrate water on the OEC is in 1:0:1:2 stoichiometry for the S_0_ → S_1_ → S_2_ → S_3_ → S_0_ transition (Suzuki et al., 2009). One of the important issues is the exact location of these active water molecules detected by FTIR difference spectra on the OEC (e.g., associated with Mn or Ca) in the new XRD structure.

## Mn LIGAND AND Mn-SUBSTRATE VIBRATION MODES IN THE LOW-FREQUENCY REGION (<1000 cm^–1^) OF THE OEC

**FIGURE 2:**
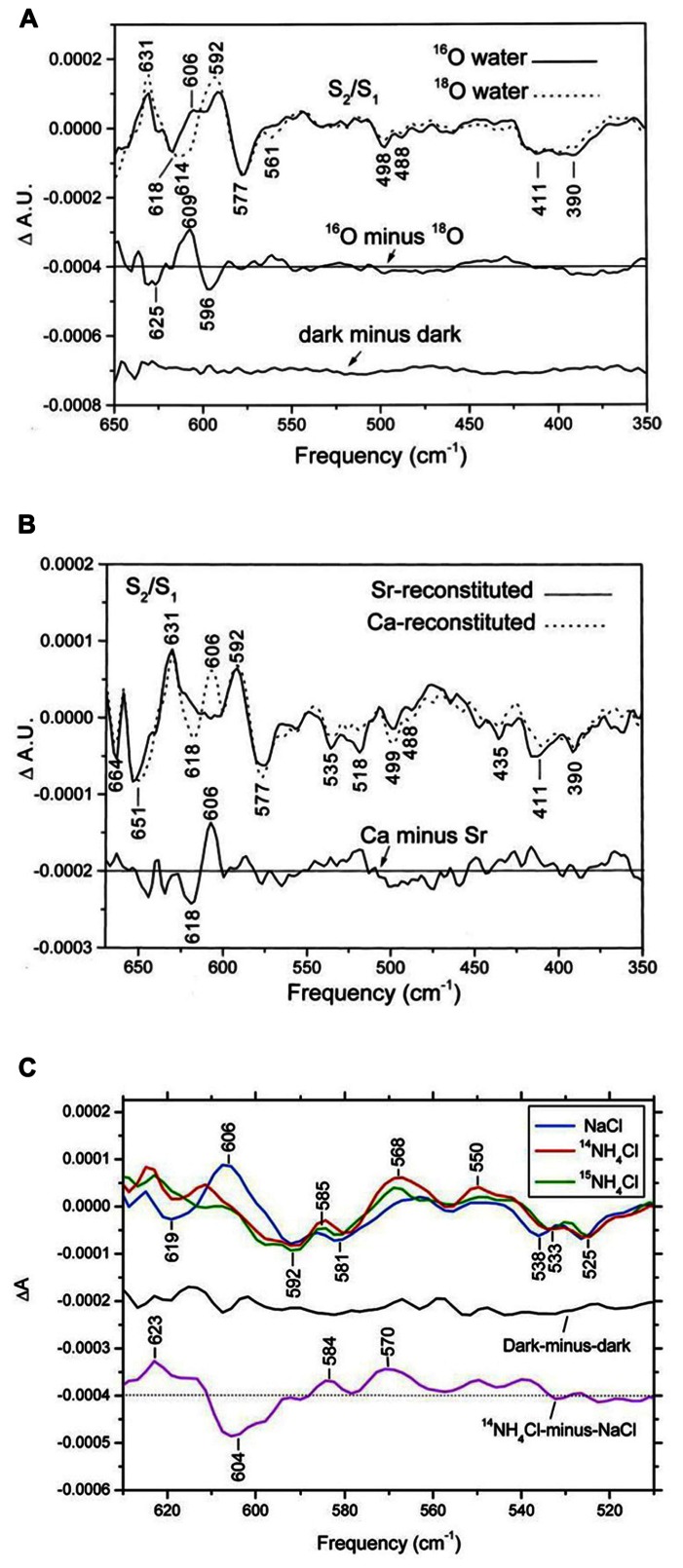
**Comparison of the low-frequency S_**2**_/S_**1**_ FTIR difference spectra of spinach PSII core complexes **(A)** in buffered H_**2**_^**16**^O (solid line) or H_**2**_^**18**^O (dotted line), **(B)** with Sr^**2+**^-reconstitution (solid line) or Ca^**2+**^-reconstitution (dashed line), and **(C)** S_**2**_Q_**A**_^**–**^/S_**1**_Q_**A**_ FTIR difference spectra with 50 mM NaCl (blue line), 50 mM ^**14**^NH_**4**_Cl (red line), or 50 mM ^**15**^NH_**4**_Cl (green line)**. The sample temperature was 250 K (reprinted with permission from Chu et al., 2000c, copyright 2000 and 2011, by the American Chemical Society).

From studies of Mn model compound, Mn-ligand and Mn-substrate vibration modes of the PSII/OEC are expected to show up in the low-frequency region (<1000 cm^-1^) of the IR spectrum (Chu et al., 2001c). In low-frequency S_2_/S_1_ FTIR difference spectra of octyl-β-D-thioglucopyranoside (OTG) PSII core preparations of spinach, a positive mode at 606 cm^-1^ in ^16^O water clearly downshifted to 596 cm^-1^ in ^18^O water (Chu et al., 2000c; **Figure 2A**). With double-difference (S_2_/S_1_ and ^16^O minus ^18^O) spectra, the 606 cm^-1^ mode was assigned to an S_2_ mode, and a corresponding S_1_ mode at about 625 cm^-1^ was identified (Chu et al., 2000c). In addition, this 606-cm^-1^ mode was up-shifted to about 618 cm^-1^ with Sr^2+^ substitution but not significantly affected by ^44^Ca isotope substitution (Chu et al., 2000c; Kimura et al., 2005a; **Figure 2B**). From these results and studies of Mn model compounds, this vibrational mode at 606 cm^-1^ in the S_2_ state was assigned to a Mn–O–Mn cluster vibration in the OEC (Chu et al., 2000c). The structure of this Mn–O–Mn cluster very likely includes additional oxo and carboxylate bridges(s). IR modes for υ(Mn=O) and υ_asy_(Mn–O–Mn) for a singly oxo-bridged Mn cluster usually occur at >700 cm^-1^ and typically have a 30–40 cm^-1^ downshift (Chu et al., 2001c). They are unlikely to be the origin of the 606-cm^-1^ mode. Furthermore, this 606-cm^-1^ mode was altered in S_2_/S_1_ FTIR difference spectra of Ala344D1Gly, Glu189Gln, and Asp170HisD1 *Synechocystis* mutant PSII particles (Chu et al., 2001a; Mizusawa et al., 2004; Kimura et al., 2005c). All the above amino acid residues are direct ligands for the Mn_4_Ca cluster. Therefore, the structure of the Mn–O–Mn cluster is structurally coupled to its surrounding ligand environment.

Low-frequency S_3_/S_2_ spectra were reported in OTG PSII core preparations of spinach, in which intense bands at 604(-) and 621 (+) cm^-1^ were sensitive to ^18^O water exchange (Chu et al., 2001b). The S_3_ mode at ~621 cm^-1^ was attributed to the Mn–O–Mn cluster mode of the S_3_ state. Kimura et al. (2005b) reported on ^16^O/^18^O- and/or H/D water-sensitive low-frequency vibrations of the OEC during the complete S-state cycle in PSII core particles from *T. elongatus*. The S_2_ mode at ~606 cm^-1^ changed their sign and intensity during S-state cycling, which indicates S-state-dependent changes in the core structure of the Mn_4_CaO_5_ cluster. In addition, several IR bands sensitive to both ^16^O/^18^O and H/D exchanges were attributed to S-state intermediates during the S-state cycling (Kimura et al., 2005b). Furthermore, an intense 577(-) cm^-1^ band in the S_2_/S_1_ spectra was found insensitive to universal ^15^N- and ^13^C-isotope labeling and assigned to the skeletal vibration of the Mn cluster or stretching vibrational modes of the Mn ligand (Kimura et al., 2003).

Low-frequency FTIR results demonstrate that one bridged oxygen atom in the Mn–O–Mn cluster of the OEC is accessible to and can be exchanged with bulky-phase water. This exchange occurs within minutes or faster because it is complete within 30 min (Chu et al., 2000c). A recent study involving W-band ^17^O electron–electron double resonance-detected nuclear magnetic resonance (NMR) spectroscopy reported that one μ-oxo bridge of the OEC can exchange with H_2_^17^O on a time scale (≤15 s) similar to that of substrate water on the OEC (Rapatskiy et al., 2012). This study also suggested that the exchangeable μ-oxo bridge links the outer Mn to the Mn_3_O_3_Ca open-cuboidal unit (O_4_ and O_5_ in **Figure 1**). The authors of this study favored the Ca-linked O_5_ oxygen assignment (Rapatskiy et al., 2012). Low-frequency FTIR results showed that the Mn–O–Mn cluster mode at 606 cm^-1^ is sensitive to Sr^2+^ substitution but not ^44^Ca substitution (Chu et al., 2000c; Kimura et al., 2005a). Considering the structure of O_5_ in the Mn_4_CaO_5_ cluster (Umena et al., 2011; **Figure 1B**), the ^44^Ca-induced isotopic shift of the Mn–O–Mn cluster mode may have been too small to be detected by previous FTIR studies. Thus, the O_5_-bridging oxygen atom is a good candidate for the exchangeable-bridged oxygen atom in the Mn–O–Mn cluster identified by FTIR. A recent continue-wave Q-band electron nuclear double resonance (ENDOR) study reported a much slower ^17^O exchange rate (on the time scale of hours) with ^17^O-labeled water into the μ-oxo bridge of the OEC (McConnell et al., 2011). Future study is required to resolve this discrepancy.

## EFFECT OF AMMONIA ON THE OEC

Because of the structural similarity between NH_3_ and H_2_O and the ability of NH_3_ to inhibit photosynthetic water oxidation, the NH_3_ binding site on the OEC might occur at the substrate water-binding site. Previous EPR studies of NH_3_-treated PSII samples demonstrated that the S_2_-state multiline EPR signal is altered when samples illuminated at 200 K are subsequently “annealed” above 250 K (Beck et al., 1986; Britt et al., 1989). FTIR studies showed that NH_3_ induced characteristic spectral changes in the S_2_/S_1_ spectra at 250 K (Chu et al., 2004a; Fang et al., 2005). Among them, the S_2_-state symmetric carboxylate stretching mode at 1365 cm^-1^ in the S_2_/S_1_ spectrum of control samples up-shifted to ~1379 cm^-1^ in NH_3_-treated samples. This carboxylate mode was also altered by Sr^2+^ substitution (Strickler et al., 2005; Suzuki et al., 2006), which indicates that the action site of NH_3_ on the OEC is near the Ca^2+^ site. In addition, the conditions that give rise to the NH_3_-induced up-shift of this S_2_-state carboxylate stretching mode at 1365 cm^-1^ are strongly correlated with those producing the modified S_2_-state multiline EPR signal (Chu et al., 2004a; Fang et al., 2005). Furthermore, a recent FTIR result showed that NH_3_ did not replace the active water molecule connected to the OEC during the S_1_-to-S_2_ transition at 250 K, whereas the Mn–O–Mn cluster vibrational mode at 606 cm^-1^ was diminished or underwent a large shift (Hou et al., 2011; **Figure 2C**). The above results are consistent with the proposal that NH_3_ may replace one of the bridging oxygen atoms, presumably O_5_, in the Mn_4_CaO_5_ cluster during the S_1_-to-S_2_ transition (Britt et al., 1989).

The other intriguing FTIR finding is that the effect of NH_3_-induced up-shift of 1365 cm^-1^ mode in the S_2_/S_1_ spectrum was diminished at temperatures above 0°C (Huang et al., 2008). The results indicate that the interaction of NH_3_ with the OEC is attenuated at temperatures above 0°C (Huang et al., 2008). In addition, a recent FTIR study reported an inhibitory effect of the ammonium cation on the PSII/OEC at 283 K (Tsuno et al., 2011). The results suggested that the ammonium cation perturbs some carboxylate residues coupled to the Mn cluster during the S_1_-to-S_2_ transition and inhibits the oxygen evolution reaction at 283 K (Tsuno et al., 2011).

## FTIR RESULTS FOR PROTEIN LIGANDS OF THE OEC

Fourier transform infrared studies involving isotopic labeling and site-directed mutagenesis have provided a wealth of information on dynamic structural changes of the protein backbones and amino acid side-chains during the S-state transitions of the OEC (Debus, 2008; Noguchi, 2008a; Shimada et al., 2011). An isotope-edited FTIR study identified the L-[1-^13^C]alanine-sensitive symmetric carboxylate stretching modes in S_2_/S_1_ difference spectra to the α-COO^-^ group of D1-Ala344 (Chu et al., 2004b). This mode appears at ~1356 cm^-1^ in the S_1_ state and at ~1339 or ~1320 cm^-1^ in the S_2_ state in unlabeled wild-type PSII particles but not in D1-Ala344Gly and D1-Ala344Ser mutant PSII particles. These frequencies are consistent with unidentate ligation of the α-COO^-^ group of D1-Ala344 to the Mn_4_Ca cluster in both the S_1_ and S_2_ state (Chu et al., 2004b; Strickler et al., 2005). In addition, substituting Sr for Ca did not alter the symmetric carboxylate stretching modes of D1-Ala344 (Strickler et al., 2005). The results suggested that the α-COO^-^ group of D1-Ala344 did not ligate Ca. In the 1.9 Å XRD structure, the α-COO^-^ group of D1-Ala344 shows very asymmetrical bridging between Mn_2_ and Ca in the cluster, with the Mn–O distance 2.0 Å and Ca–O distance 2.6 Å (Kawakami et al., 2011). In addition, the isotopic bands for the α-COO^-^ group of D1-Ala344 showed characteristic changes during S-state cycling (Kimura et al., 2005d). These results indicated that the C-terminal Ala 344D1 is structurally coupled, presumably directly ligated, to the Mn ion that undergoes oxidation of Mn(III) to Mn(IV) during the S_1_-to-S_2_ transition and is reduced in reverse with the S_3_-to-S_0_ transition (Chu et al., 2004b; Kimura et al., 2005d). In contrast, mutations of D1-Asp170, D1-Glu189, and D1-Asp342 did not eliminate any carboxylate vibrational stretching modes during S-state cycling of the OEC (Debus et al., 2005; Strickler et al., 2006, 2007). Recent computational studies suggested that vibrations of carboxylate ligands can be quite insensitive to Mn oxidation, if they are not coordinated along the Jahn–Teller axis (Sproviero et al., 2008). In their model, the only amino acid residue that is ligated along the Jahn–Teller axis of a Mn^*III*^ ion is CP43-E354.

Of note, CP43-E354Q mutant PSII particles gave rise to characteristic spectral changes in the amide and carboxylate stretch regions of FTIR difference spectra during S-state transitions (Strickler et al., 2008; Shimada et al., 2009; Service et al., 2010). In addition, the weakly H-bonded O–H stretching modes of the active water molecule associated with the OEC were significantly altered in S_2_/S_1_ FTIR difference spectra of CP43-E354Q mutant PSII particles (Shimada et al., 2009). Furthermore, H_2_^18^O exchange mass spectrometry experiments showed that the CP43-E354Q mutation weakened the binding of both substrate-water molecules (or water-derived ligands), particularly affecting the one with faster exchange in the S_3_ state (Service et al., 2010). The XRD structure of the OEC showed that coordinated water molecules were on Ca^2+^ and Mn_4_, which were both not ligated by CP43-E354 (Umena et al., 2011). Presumably, CP43-E354Q mutation may induce significant structural changes to the Mn_4_CaO_5_ core that affects associated active water molecule(s) on the OEC during the S_1_-to-S_2_ transition.

A recent time-resolved infrared study revealed the proton and protein dynamics associated with the OEC during the S-state transitions (Noguchi et al., 2012). The results suggest that during the S_3_-to-S_0_ transition, protons are greatly rearranged to form a transient state before the oxidation of the Mn_4_CaO_5_ cluster that leads to O_2_ formation. In addition, an early proton movement was detected during the S_2_ → S_3_ transition, indicating a proton release coupled with the electron transfer reaction. Furthermore, a relatively slow carboxylate movement occurred in the S_0_ → S_1_ transition, which might reflect the protein relaxation process to stabilize the S_1_ state (Noguchi et al., 2012). This study demonstrates that time-resolved infrared technique is extremely useful to monitor proton and protein dynamics of the OEC during photosynthetic oxygen evolution.

## BIOINORGANIC MODELS FOR FTIR SPECTRAL INTERPRETATION

Vibrational data from model compounds relevant to the OEC is crucial to interpret FTIR data of the OEC during S-state cycling. However, vibrational data for synthetic multinuclear Mn complexes are still limited (Cua et al., 2003; Berggren et al., 2012). Particularly, vibrational data are needed for the Ca–Mn multinuclear cluster that models the Mn_4_CaO_5_ cluster (Kanady et al., 2011; Mukherjee et al., 2012). One previous study reported IR spectra and normal mode analysis of the adamantine-like complex [Mn_4_O_6_(bpea)_4_]^n+^ (Visser et al., 2002). By using the electrochemical method to record the difference IR spectrum and ^18^O isotopic labeling, the authors identified Mn–O vibrational modes for [Mn^IV^_4_] and [Mn^IV^_3_Mn^III^]. Comparison with OEC data ruled out the adamantine-like complex as the possible structure intermediate. Nevertheless, this approach is very powerful for interpreting FTIR data for the OEC during S-state cycling.

## CONCLUSIONS AND PERSPECTIVES

Light-induced FTIR difference spectroscopy has become a fruitful structural technique to study the molecular mechanism of photosynthetic water oxidation. The new high-resolution XRD structure of the OEC has served as a crucial foundation for designing FTIR experiments and interpreting FTIR data. Combined with isotopic labeling, site-directed mutagenesis, model compound studies, and normal mode analysis, FTIR difference spectroscopy will continue to provide important structural and mechanistic insights into the water-splitting process in PSII.

## Conflict of Interest Statement

The author declares that the research was conducted in the absence of any commercial or financial relationships that could be construed as a potential conflict of interest.

## Abbreviations

EPR: electron paramagnetic resonance
FTIR: Fourier transform infrared
OEC: oxygen-evolving complex
OTG: octyl-β-D-thioglucopyranoside
PSII: photosystem II
Q_A_: primary quinone electron acceptor in PSII
XRD: X-ray diffraction
